# Cat and Mouse: HIV Transcription in Latency, Immune Evasion and Cure/Remission Strategies

**DOI:** 10.3390/v11030269

**Published:** 2019-03-18

**Authors:** Aurélie Delannoy, Mikaël Poirier, Brendan Bell

**Affiliations:** Département de Microbiologie et Infectiologie, Faculté de Médecine et des Sciences de la Santé and Centre de recherche du CHUS, Université de Sherbrooke, Sherbrooke, QC J1E 4K8, Canada; Aurelie.Delannoy@USherbrooke.ca (A.D.); Mikael.Poirier@USherbrooke.ca (M.P.)

**Keywords:** HIV, latency, transcription, cure, remission, immune evasion, immunotherapy

## Abstract

There is broad scientific and societal consensus that finding a cure for HIV infection must be pursued. The major barrier to achieving a cure for HIV/AIDS is the capacity of the HIV virus to avoid both immune surveillance and current antiretroviral therapy (ART) by rapidly establishing latently infected cell populations, termed latent reservoirs. Here, we provide an overview of the rapidly evolving field of HIV cure/remission research, highlighting recent progress and ongoing challenges in the understanding of HIV reservoirs, the role of HIV transcription in latency and immune evasion. We review the major approaches towards a cure that are currently being explored and further argue that small molecules that inhibit HIV transcription, and therefore uncouple HIV gene expression from signals sent by the host immune response, might be a particularly promising approach to attain a cure or remission. We emphasize that a better understanding of the game of “cat and mouse” between the host immune system and the HIV virus is a crucial knowledge gap to be filled in both cure and vaccine research.

## 1. Introduction

Human immunodeficiency virus (HIV-1) virus was identified in 1983 as the cause of the acquired immunodeficiency syndrome (AIDS) [1]. The discovery of HIV and its subsequent characterization made possible the development of the antiretroviral therapy (ART) currently used clinically to treat HIV infected people. ART is a combination of drugs that targets the replication of the virus and decreases HIV viremia durably in patients, curtails transmission and extends life. However, the disease is far from being under control. In 2017, a report of UNAIDS estimated that 77.3 million people had been infected worldwide since the start of the epidemy, and that 59% of the 36.9 million people who are still living with the virus had access to the treatment [2]. This constitutes a significant progress since only half of them had access to a treatment in 2010, but there is still a lot to do to achieve the 90-90-90 targets by 2020 (90% of people living with HIV know their status, 90% of them are on HIV treatment, among whom 90% achieve viral suppression). Moreover, even in regions where this goal was almost reached in 2016 [3], there is still a high need for new therapeutic approaches that could definitely cure the disease. The burdensome side effects of ART and the possible emergence of resistance to available therapy are two reasons to pursue a cure [4,5].

## 2. Latency and the Latent Reservoirs

The main obstacle to a cure resides in the so-called latent reservoirs, generally defined as cells bearing full length HIV-1 proviruses that are replication competent but transcriptionally inactive. These cell populations are not targeted by ART, either because of their latent status or because they cannot be reached by the drugs (reviewed in [6,7]) but can still be reactivated upon treatment interruption [8].

### 2.1. The Latent Reservoir Is Constituted of Several Subsets of Immune Cells with Specific Features

The latent reservoir is established very early upon HIV infection and although treatment with ART during the acute phase can reduce the size of the reservoir, it is not sufficient to prevent the establishment of a pool of latently infected cells [9,10]. It has often been assumed that viral latency occurs when recently infected activated CD4+ T cells revert to a memory state [6]. However, accumulating evidence suggests that the latent reservoir is composed of a more diverse population and that its establishment and dissemination are regulated by more complex mechanisms.

#### 2.1.1. The Different Populations of the Reservoir

The largest fraction of the reservoir is constituted by memory T cells (reviewed in [11]) These memory T cells can be divided into several subsets with distinct features (Reviewed in [11,12]). T stem cell memory (T_SCM_) are the least differentiated subset and are the precursors of central memory (T_CM_), effector memory (T_EM_) and terminally differentiated T cells (T_TD_). The transitional memory (T_TM_) are an intermediate between T_CM_ and T_EM_ and effector cells. Migratory memory (T_MM_) cells circulate between the peripheral blood compartment and the tissues where another subset, the tissue resident memory (T_RM_), stay more permanently (reviewed in [12]). Other T cell types are involved in the maintenance of the reservoirs, e.g., the naïve T cells (T_NA_), the precursors of T_CM_, which are permissive to HIV but at low frequencies [13,14]. All these subsets contribute to the reservoir but to different extents (reviewed in [12]), and this contribution is mostly affected by the maturation and differentiation state of the cell [11]. It was first shown that, among the T cell population of the reservoir, central memory T cells, was the most prevalent subset [6,13,15], while the T_TD_ was the least prevalent one [6]. This is due to their life span, which is longer than the one of the effector or terminally differentiated subsets [16], and their IL-7 driven homeostatic proliferation [17]. More recently, T_SCM_ were shown to persist even longer, with an average life span of 277 month (vs. 144 for central memory T cells) and a more pronounced proliferation capacity [18,19]. These features may explain the increased overrepresentation of this subset in the total reservoir compared to central memory T cells along the duration of ART [18,19]. The undifferentiated state also appears to correlate with the permissiveness to the virus. T_SCM_ have been shown to be highly permissive since the proportion of cells containing HIV-1 DNA is higher in this subset than in T_NA_, T_CM_, T_EM_ and T_TD_ [19].

The above findings are in accordance with the observation that Th17 polarized cells are more susceptible to HIV-1 infection [20]. Interestingly, these cells have stem-cell-like properties since they have a long life span, replicate in an homeostatic manner and have a wide developmental plasticity [21,22]. In addition, a subset of the polarized Th1/Th17 CD4+ effector T cells, containing replication competent HIV-1, has been shown to survive for several years upon ART [23]. A recent study by Wacleche et al. demonstrated that a restricted subpopulation of Th17 cells expressing the marker CCR6, but not CCR4 or CXCR3, was predominant in the blood and the lymph nodes of ART treated individuals compared to the other Th17 subsets [24]. Interestingly, this specific subset shows features that are characteristic of follicular T helper cells (Tfh), and thus could be an intermediate between Th17 and Tfh. Follicular T helper cells are T helper cells that are located in the lymph nodes and they have also been identified as a possible component of the reservoir since they are highly permissive to the virus and are a major site of HIV replication in untreated patients [25]. Moreover, a subpopulation of circulating T cells that share properties with Tfh, subsequently named peripheral follicular T helper cells (pTfh), have been identified recently [26]. The contribution of Tfh and pTfh to the reservoir is still unclear and under investigation, but a study showed that HIV-1 DNA was detected in pTfh of the majority of the patients [23]. These cells even represent 20% of total memory T cells, are more permissive to HIV-1 infection and carry a higher proportion of replication competent proviruses than other T_CM_ [26,27,28]. Garcia and coworkers have demonstrated that the largest difference in reservoir size between elite controllers and patients treated with successful ART was observed in pTfh subset. This finding suggests that pTfh subpopulation may be a key actor in the maintenance of the reservoir, likely because of its ability to support high levels of HIV replication [29]. 

Finally, an atypical subset of the T cell lineage, the γδT cells, that have the ability to recognize bacterial pathogens but can also display a memory phenotype, have been shown to carry latent HIV-1 proviruses and could then be part of the reservoir [30].

This review focuses on CD4+ T cells because they constitute the most extensively studied reservoir [31,32]. However, it is worth mentioning that other populations of the immune system, such as monocytes/macrophages, natural killer or dendritic cells can also contribute to the reservoir in a potentially significant manner. Monocytes/macrophages express the CD4 receptor, although at lower levels compared to CD4+ T cells and, therefore, are permissive to HIV-1 infection [33]. Moreover, macrophages are resistant to the cytopathic effects of the virus and show long lifespans [34,35]. Therefore, the macrophages might contribute significantly to the reservoir by sustaining productive infection and facilitating new infection of CD4+ T cells [36,37]. The macrophages are also involved in the infection of the central nervous system since infected macrophages have been detected in the brain of long term ART treated individuals [38]. On the other hand, mature dendritic cells are potently resistant to HIV-1 infection, notably through the high expression of the host restriction factor SamHD1, so only a small fraction of the dendritic cell population is efficiently infected by HIV [36,37]. However, dendritic cells retain viral particles at their surface that can remain infectious for several months [39] and may contribute to the new infection of CD4+ T cells [40]. 

#### 2.1.2. The Different Compartments of the Reservoir

The cells that constitute the reservoir are present in different compartments, including the peripheral blood, the central nervous system, the lymph nodes, the gut associated lymphoid tissue (GALT), and, more generally, tissues that contain HIV-infected cells [33,41,42,43]. The peripheral blood compartment is the best characterized and the most extensively studied for feasibility reasons. However, increasing evidence suggests that the other compartments may also contribute to the HIV-1 reservoirs, especially the GALT [43]. The GALT is the largest lymphoid tissue in the body and shows a 2–4-fold increase in the proportion of cells containing HIV-1 DNA than the peripheral blood compartment [43]. Two subsets of memory T cells have been shown to contribute specifically in the compartments: the tissue resident memory T cells (T_RM_), which stay in a specific region of a tissue, and the migratory memory T cells (T_MM_) that can get back to the blood circulation [41,44,45]. Together with the macrophages, these T_MM,_ can migrate between the blood and the tissues and might participate in the dissemination of the virus and the establishment of reservoirs in the different compartments [43,46]. 

The lymph nodes represent a challenge for an HIV cure for three main reasons. Firstly, they are home to follicular T helper cells (Tfh), a subset of T_CM_ that are the major contributor to latency persistence in ART-treated or controller patients [47,48]. Secondly, they promote the infection of those Tfh by keeping them in close proximity with the follicular dendritic cells within structures called B-cell follicles [25,49]. Thirdly, they have been shown to be less accessible to drugs than blood cells [50]. The recent identification of pTfh might help further characterize the Tfh reservoir.

Two other HIV sanctuaries are the genital tract and the brain [38,51]. In the testes, the reservoir is mainly constituted of CD4+ resting T cells, whereas in the brain most of the latently infected cells are macrophages [38,51]. Both present the same obstacle: a tight barrier that separates them from the blood stream and can impair the distribution of ART into these organs [50]. A recent study by Huang et al. has shed the light on the mechanisms that control the selective permeability of the blood–testis barrier to certain molecules of ART in human biopsies [52]. The study characterized the expression and the localization of ABC and SLC drug transporters, as well as metabolic enzymes and nuclear receptors previously shown to be involved in antiretroviral drug penetration in biopsies of ART treated HIV infected patients and uninfected individuals. They also showed that drug penetration was highly variable from one drug to the other. For example, nucleoside/nucleotide reverse transcriptase inhibitors (NRTIs) showed levels comparable to plasma, whereas protease inhibitors had diverse penetration with darunavir concentrations falling below therapeutic values. 

#### 2.1.3. Establishment and Maintenance of the Reservoir

It has long been thought that memory T cells had a long life span (up to several years), mostly based on the observation that the immune memory itself can last for years (reviewed in [53,54]). However, data now support the notion that the life span of memory T cells is shorter (from six months to one year) and imply that the pool of latently infected memory T cells is maintained by mechanisms other than the simple longevity of latent T cell reservoirs (reviewed in [6,53,55]). Some of these subsets are maintained by proliferation, since T_CM_ and T_TM_ cells expressing higher levels of proliferation markers ki67 and PD1 are enriched for HIV-1 provirus in ART treated individuals [6]. This hypothesis is also supported by the homogeneity of the viral DNA sequences in the reservoir of patients on ART treatment [56,57,58]. The recent observation that CD4+ T cells can undergo homeostatic proliferation without necessarily reactivating viral expression [59], and that latently infected cells might need more than a single round of stimulation to be reactivated [60,61,62] supports this notion. Memory T cells are permissive, albeit at low frequency, to HIV infection [28,63] and the virus can be transmitted efficiently by cell-to-cell contact, either between T cell and antigen presenting cells (e.g., macrophages or dendritic cells) or even between two T cells [55,64,65]. A recent study by Agosto et al. showed that activated HIV infected CD4+ T cells have the ability to infect resting CD4+ T cells by cell-to-cell contact and that this mode of infection generated latent viruses with a decreased reactivation ability compared to other modes of infection [55]. These observations suggest that viral expression might be less stochastic and more tightly regulated than it was assumed previously. 

### 2.2. The Establishment and Maintenance of the Latency Involves the Hijacking of Different Cellular Processes

The quantification and characterization of the reservoir has long been a challenge due to the weak proportion of replication competent proviruses among the total population of HIV-1 infected cells. To date, the quantification of the reservoir has mainly used two techniques: qPCR targeting the viral DNA and quantitative viral outgrowth assay (qVOA) [66]. The quantification by qPCR is very sensitive but has been shown to overestimate the reservoir [61,67,68,69]. On the other hand, qVOA might underestimate the size of the reservoir since some of the latent integrated proviruses require several rounds of stimulation before reactivation [60,61,62]. The recent development of new single cell-based methods should provide more accurate measurements of the latent reservoir. One new technique called flow-FISH combines the detection of GagPol mRNA by in Situ hybridization with antibody staining of Gag protein and allows the quantification and characterization of the reservoir in clinical samples [70]. Another example is the use of a dual-fluorescence based reporter vector combined to cell sorting on the base of their ability to express the virus and OMICS analysis of the different subsets shed the light on the mechanisms governing HIV latency [71,72,73]. The identification of 70 potential biomarkers of latency by an integrative analysis of mRNA expression and epigenetic marks has also facilitated a better characterization of latent pools [74]. 

#### 2.2.1. HIV Plays a Game of Cat and Mouse

Several lines of evidence point toward HIV latency as an evolutionarily acquired advantage to escape the immune system. HIV can also manipulate several components of immune signaling, presumably to its own advantage. An example is the PD1 negative regulator of the immune response [75,76] that plays a role in HIV infection and latency. It has been shown that HIV-1 preferentially infects T cells that express high levels of PD1 [6,77] and that this protein is found upregulated at the surface of both HIV-specific CD8+ and CD4+ T cells [78,79,80,81]. Moreover, a recent study by Evans et al. has shown that the expression of PD1 and other immune checkpoints favors HIV latency in resting CD4+ T cells and that the use of an antibody targeting PD1 can reactivate HIV-1 expression in an individual under ART [82]. Finally, it has been demonstrated that another way for HIV to neutralize the immune system is to target preferentially HIV-specific CD4+ T cells [83].

#### 2.2.2. The Hypermutagenicity of the Virus Favors the Latency

It is estimated that only 5% of latent HIV proviruses are replication competent [68,84]. The remaining 95% were shown to carry deleterious modifications of their genome such as large deletions or multiple mutations [72]. The mutation rate of HIV-1 has been evaluated at 4.1 × 10^3^ per base per cell in vivo, the highest of any biological entity [85], and is mainly due to the error-prone HIV-1 reverse transcriptase as well as G-to-A APOBEC3G induced hypermutation [86,87]. APOBEC proteins are restriction factors involved in innate immunity with a cytidine deaminase activity and are inhibited by the viral accessory protein Vif [88]. This high mutation rate is a solid advantage for the rapid evolution and adaptability of the virus and could then promote HIV latency under the selective pressure imposed by ART treatment [89]. Evidence of the impact of mutations on latency has been provided by a recent study using a dual fluorescence reporter vector to identify seven reactivatable latently infected clones, among which five carried mutations. Interestingly, two of these clones had mutations in the *trans*-activation response element (TAR), disrupting the interaction with the viral *trans*-activator of transcription Tat [72]. Mutations in the viral genome occur most likely before initiation of ART, as suggested by the decay in genetic diversity of the virus observed after ART initiation and the capacity of latently infected CD4+ T cells to expand clonally [90,91].

#### 2.2.3. The Site of Integration and the Epigenetic Marks Play a Role in HIV Expression

The site of integration of the HIV provirus into its host genome seems to play an important role for HIV expression and thus for latency regulation. Large scale sequencing studies performed in several model cell lines have identified millions of possible integration sites throughout the genome [92,93] and even if a consensus sequence has been identified, it modestly impacts the choice of the integration site in vitro [94]. However, the virus targets specifically regions of the genome that are actively transcribed (reviewed in [95,96]) and carry epigenetic marks associated with an open chromatin and active transcription such as CpG islands, acetylation of H3K9 or trimethylation of H3K36 [97,98,99]. HIV has also been shown to preferentially integrate into genes that undergo alternative splicing, without any preference for exons or introns in HEK293T [92]. This choice can be explained by the molecular mechanisms that underlie the integration. HIV enters the host cell nucleus as a nucleoprotein complex (called the pre-integration complex) composed of the full length viral DNA genome, viral proteins such as Vpr or the integrase and host cell factors. The integrase then interacts with the lens epithelium-derived growth factor (LEDGF) and the cleavage and polyadenylation specificity factor 6 (CPSF6). LEDGF binds trimethylated H3K36, an epigenetic mark associated with active transcription [100] as well as splicing factors [92], and CPSF6 is also involved in splicing [97]. The physical interactions between the preintegration complex and factors involved in splicing explains the preference for integration in highly expressed and spliced genes. Finally, the nuclear organization might also have an important role in the choice of the integration site. Indeed, pre-integration complexes have been detected in areas of decondensed chromatin at the periphery of the nucleus [99], and HIV recurrent integration genes (RIGs) were found clustered in regions of the chromosomes that are close to the nuclear pore complexes (NPC) [98]. 

Taken together, the above evidence points towards an important role of the genomic context in the expression pattern of the integrated provirus and it is tempting to assume that the site of integration could predict the latency status. However, the data available to date do not unambiguously support a simple determinant role for the genomic integration site on latency. For example, it has been observed that the integration of HIV in alphoid repeats or into gene deserts might favor latency [101,102] and that viral transcription was dependent on the orientation of the integration and the transcription rate of the host gene in primary and model cell lines [103,104]. Moreover, a recent study using a dual fluorescent reporter system in primary CD4+ T cells showed that the reactivation potential of latently infected cells correlates with gene transcription and that non-reactivable proviruses were integrated to a higher extent in heterochromatic regions associated with the lamins [73]. On the other hand, a meta-analysis performed in five model cell lines failed to find a correlation between latency and epigenetic marks, the orientation of integration or the presence of alphoid repeats [101]. Another model was proposed by Weinberger et al., based on mathematical modeling, wherein the latency phenotype would be primarily determined by the stochastic expression of the Tat protein in addition to the genomic environment, cell signals or the cell cycle [105].

Overall, the available data suggest that the site of integration or the chromatin status might not be the only mechanism explaining the latency establishment and/or regulation and that it would be better to target other components that are less dependent on genomic integration site or chromatin state.

#### 2.2.4. RNA Processing

RNA processing includes alternative splicing, 5′ capping, 3′ end polyadenylation, RNA modification and quality control, all of which are critical for HIV protein expression.

The 9 kb genome of the virus encodes nine genes whose sequences partially overlap. To ensure proper gene expression, the virus hijacks the splicing system of the host cell and produces up to 70 different RNA isoforms [106]. The splicing of HIV is regulated by sequence motifs called splicing enhancers and splicing inhibitors, as well as stem loop structures on the RNA which can be bound by host cell factors of the SR and hnRNP family (reviewed in [107]). Thus, the binding of SRp75 or SRP40 and ASF/SF2 on specific enhancers promote splicing towards Vif or Nef, respectively [108,109], and SRSF10 is required for the efficient expression of Tat, Gag and Env [110]. On the other hand, hnRNP A1 and hnRNP H binding to splicing silencers prevents Tat expression [111,112]. Several lines of evidence also suggest that, during the later stages of the HIV life cycle, alternative splicing is repressed to promote the production of full-length and unspliced HIV RNA [113] and that Tat has a central role in this process. Indeed, Tat promotes HIV mRNA splicing via the phosphorylation of SF2 [113]. However, as the replication cycle progresses, Tat becomes acetylated and ends up repressing splicing through the recruitment of the splicing inhibitor p32 [113]. Other accessory proteins of HIV have been shown to regulate splicing. For example, Vpr has the ability to repress splicing [114].

Other mechanisms of RNA metabolism are also important for the viral replication. For example, Sam68 is involved in the polyadenylation of the HIV mRNA [115], hnRNP E1 regulates Rev expression through inhibition of RNA translation [116] and Rev itself is involved in mRNA translation and required for the nuclear export of unspliced RNA [117]. The RNA quality control pathway is also involved since UPF1 has been shown to favor reactivation, whereas UPF2 and SMG6 repress it [118].

Direct evidence now suggests that RNA processing impacts latency and the reactivation potential. Two recent studies using droplet digital PCR (ddPCR) to characterize patient latently infected cells have shown a defect in transcriptional regulation as well as in the splicing [119,120]. Moreover, a study by Golumbeanu et al. used single cell RNA sequencing to show that the reactivation potential of latently infected cells can be explained by the differential expression of 134 genes enriched for RNA metabolism related pathways such as RNA splicing, Ribosomal genes or translational regulation [121]. It has also been observed that the transcriptional activation induced by Latency reactivating agents (LRA) such as HDAC inhibitors was not necessarily correlated with protein expression in vivo [122], probably because these LRAs fail to reactivate factors required for RNA processing such as the Rev cofactor MATR3 [123].

#### 2.2.5. miRNAs

MicroRNAs (miRNAs) are short single stranded oligonucleotides that specifically address the RNA-induced silencing complex (RISC) to mRNA to mediate post transcriptional gene silencing [124]. Although the impact of miRNA mediated regulation of HIV is an emerging topic, an accumulation of evidence suggests that this mechanism might be important in several ways. Several of these miRNAs have been shown to modulate HIV expression (Table S1) [125,126,127,128]. These miRNAs can directly target the virus, such as miR-29a and miR-125b, that target *nef* or the 3′UTR sequence respectively (Table S1) [126,128]. Other miRNAs can regulate HIV indirectly by targeting cellular factors. For example, miR-17/92, miR-17-5p and miR-20a downregulate the Tat co-activator PCAF and miR-29b, miR-150, miR-223 and miR-27b inhibit the expression of Cyclin T1 (Table S1) [127,129,130]. RNA silencing machinery inhibits HIV replication and the viral proteins Tat and Vpr inhibit RNA silencing [129,131,132]. The HIV-1 virus itself encodes for miRNAs that target either host cell factors, such as the anti-apoptotic protein AATF or HIV itself. Interestingly, several HIV-encoded miRNAs have been involved in HIV transcriptional regulation and act at the level of the 5′ LTR. MiR-M367 impairs viral expression and targets the U3 negative response element in CD4+ T cell lines (Table S1) [133]. TAR-derived miRNAs exert the same effect through the chromatin remodeling of the promoter by HDAC-1 [134]. Mir-H3, on the other hand, promotes HIV transcription when overexpressed and targets the TATA box in activated primary CD4+ T cells [135]. Cellular miRNA expression profiles can be modified upon HIV infection [136] and CD4+ T cell activation [128,137]. More importantly, it has been shown that elite controllers show higher plasma levels of several miRNAs that can reduce HIV infection in vitro [138]. Those miRNAs could then be an attractive way to detect and diagnose HIV. Similar to the endogenous targeting of HIV by miRNAs, it may be possible to exploit exogenous RNA-based strategies for HIV cure strategies. For example, shRNA and siRNA have been shown to target and efficiently silence HIV-1 expression [139,140,141]

#### 2.2.6. The Cell Cycle

The regulation of the host cell cycle and of the viral replication are tightly linked in CD4+ T cells. This was first suggested by the observation that cell cycle activation is required for HIV replication [142,143]. A growing body of evidence has now identified multiple viral strategies to hijack cell cycle regulation. For example, the promotion of transcription by Tat *trans*-activation is dependent upon cell cycle regulation since it depends mostly on the expression and activity of cyclin T1 and cyclin dependent kinases. It has been shown that the downregulation of Cyclin T1 is required for the establishment of latency [144] and that this downregulation is mediated by the miRNAs miR-27b, miR-150 and miR-223 in resting T cells (Table S1) [127]. In addition, Cdk9 kinase activity, which is required for HIV transcription elongation, is regulated in a cell cycle dependent manner through dephosphorylation by PPM1A [145] and phosphorylation by Cdk7 [146]. The virus can also manipulate the cell cycle with the viral accessory protein Vpr which is highly conserved throughout HIV evolution [147] and mediates cell cycle arrest in G2/M phase [148,149]. This cell cycle arrest in G2/M has been associated with an increased activity of the HIV promoter in cellulo [150,151] and in vivo [72]. Unexpectedly, the establishment of latency does not require the inactivation of Vpr [152] and MX1, a host cell factor known to be upregulated by Vpr [153] that was found overexpressed in five HIV-1 latently infected cell lines [74]. In addition, a recent study by Kok et al., using a dual fluorescence HIV-1 based vector, has shown that a subpopulation of latently infected lymphoblastic SUP-T1 cells was able to undergo reactivation in a cell cycle dependent manner. This observation was supported by the specific regulation, in these cells, of genes involved in cell cycle regulation, such as FosB, NEAT1, EGFR or RN7SK. Moreover, large fractions of those cells were in the S and G2 phase and reactivation of latent cells was achieved by blocking the cells in G2 phase through treatment with genistein or nocodazole [72]. These observations suggest that the persistence of the reservoir requires the dampening instead of the total suppression of the cell cycle dependent activation of viral expression. This could then allow clonal expansion without necessarily activating HIV replication and/or the resulting cell clearance by ART or the immune system. This hypothesis is further supported by the observation that homeostatic proliferation of T_CM_ reservoir induces a partial reactivation of HIV without cell death, and thus could account for the maintenance of the reservoir [59].

#### 2.2.7. Apoptosis

The cells that compose the latent reservoir are characterized by a long life span. This is in part due to their low proliferation rate, but several studies suggested that latency is also associated with modifications in the regulation of cell survival and apoptosis. For example, antiapoptotic factors such as Bcl-2, Mcl-1, cFlip and XIAP have been found to be overexpressed [154,155,156], whereas proapoptotic Bax and Fadd [157] have been found to be downregulated in latently infected T-cells. The regulation of these factors also prevents HIV expression. For example, ROS have been shown to induce HIV reactivation [158], but the overexpression of Bcl-2 protects the cells from ROS through their neutralization by elevated levels of glutathione and thioredoxin [154]. A role has also been proposed for caspases in HIV-1 reactivation [159] and a quantitative mass spectrometry-based study of latently infected CD4+ T cells showed that they express increased levels of BIRC5 (an inhibitor of Caspase 9) [160]. More recently, the ontological study of 70 markers of latency has shown the enrichment for processes involved in cell death inhibition [74].

Furthermore, it has been demonstrated that primary culture of HIV-1 latently infected T_CM_ have longer telomeres, suggesting that reactivation of the telomerase could contribute to the longevity and clonal expansion of the reservoir [161]. It has also been shown that the reservoirs were enriched for viral integration in the genes coding for BACH2, STAT5/SB and MLK2 and that the interruption of those genes also favors the survival and clonal expansion of those cells [162,163].

#### 2.2.8. Autophagy

The catabolic process autophagy contributes to HIV latency. HIV-1 regulates autophagy in a cell type dependent manner. In macrophages, virus replication is promoted by the early stages of autophagy, while viral replication is inhibited by the later stages [164,165]. In CD4+ lymphocytes, activation of autophagy limits the viral replicative potential [166] and HIV infected individuals have this process impaired when compared to viremia or elite controllers [167]. It has been shown that autophagy promotes latency in part through the p62-mediated selective degradation of Tat [166]. This degradation also occurs to the secreted pool of Tat that can be captured by bystander cells and is controlled by the transient induction of autophagy by the viral protein Env [166]. 

#### 2.2.9. Transcription is Linked to Most of the Processes Listed above

In this review, we focus primarily on the role of transcription in latency regulation because it is the first step in the reactivation of latent HIV and has therefore received the greatest amount of attention in the study of HIV gene expression mechanisms. In the case of host gene expression, the regulation of the transcription is tightly coupled to RNA processing mechanisms (reviewed in [168]). Since the virus uses the host cell machinery for its expression, it is not surprising that many of the processes involved in latency regulation also show connections with transcription, such as the cell cycle [72,150,151] or splicing [169,170]. A concrete example of how transcription impacts RNA processing was shown by the observation that replacing the HIV promoter by a CMV promoter modifies HIV mRNA splicing in vitro [171]. The fact that transcription is coupled to multiple downstream gene expression events is relevant in the search for drug targets that synergistically impact HIV latency by simultaneously acting on several steps in the HIV gene expression pathway.

## 3. The HIV Proximal Promoter and its Cognate Transcriptions Factors

One of the features of the HIV genome is that it contains a single promoter within the 5′ LTR that is alone responsible for driving viral transcription. The HIV promoter drives the expression of viral RNAs that, after transcription, undergo splicing, processing and transport to allow the translation of the 16 viral proteins needed for the production of new virions and the regulation of latency (reviewed in [172]).

**Figure 1:**
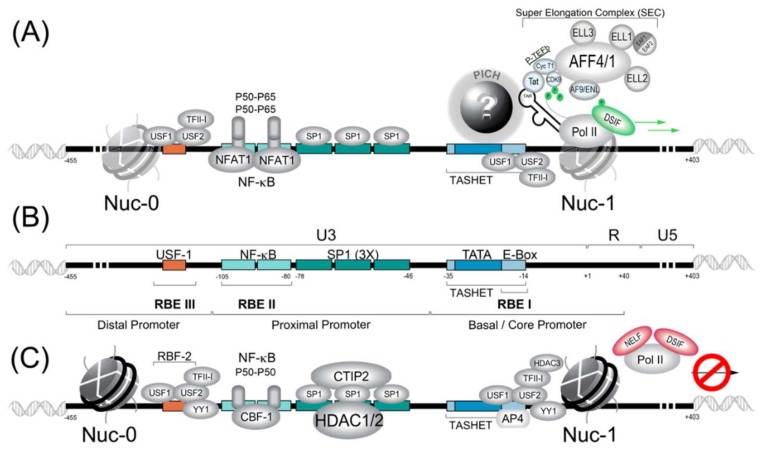
The human immunodeficiency virus (HIV) 5′ long terminal repeat (LTR) promoter region responsible for directed viral transcription is schematically represented. (**A**) The HIV LTR in its active state is depicted where host cell transcription factors and viral Tat activate transcription. **PICH** refers to **p**re-**i**nitiation **c**omplexes of **H**IV whose full identity remains an enigma. **TASHET** is the **T**ATA box and **a**djacent **s**equences of **H**IV **e**ssential for **T**at *trans*-activation. The **s**uper **e**longator **c**omplex (**SEC**) is shown at the right. (**B**) For clarity, key *cis*-acting DNA elements are shown in the absence of transcription factors. **RBE** refer to **R**BF-**b**inding **e**lements. (**C**) The HIV LTR is shown in a hypothetical repressed state associated with viral latency. **RBF**-2 refers to the **R**as-**r**esponsive region **b**inding **f**actor **2**. Note that certain transcription factors can play both positive and negative roles in HIV latency depending on the cellular context. See text for details.

The HIV core promoter contains a TATA box DNA motif that is essential for the recruitment of the pre-initiation complex, which recruits RNA polymerase II (RNAPII) (Figure 1). Downstream of the TATA box, the HIV promoter possesses an E-box (enhancer box), which is the target of the transcription factor AP4 (activating enhancer binding protein 4) (reviewed in [173]), as well as the host cell transcription factor USF1 (upstream stimulatory factor 1) [174,175]. Upstream of the TATA box within the proximal promoter, there are binding sites allowing SP1 and NF-κB transcription factors to interact with the promoter and to influence activation or repression (Figure 1).

Many host cell transcription factors have been found to interact with the HIV LTR (reviewed in [176,177]). Here, for concision, we provide a synopsis of the factors with the best-established roles in HIV transcription. Readers should bear in mind that many other factors have been implicated, and that the list of transcription factors impacting HIV latency continues to expand. We summarize the general transcription factors (GTFs), P-TEFb, NF-κB, NFAT, SP1, HDAC, AP4 and RBF-2 (USF1/USF2/TFII-I), and their roles in HIV transcription and latency. Work with cell lines has been essential for the discovery of transcription factors, as these tools provide an experimentally tractable system and suitable amounts of starting material. Thus, once transcription factors are identified, it is important to test their function in primary cells to more closely approximate the physiological situation. Work with primary cells should be facilitated by recent success with CRISPR/Cas9-based genomic editing of primary CD4+ lymphocytes [178]. Ultimately, transcription factors that bind to the HIV LTR must be verified in vivo in animal models such as humanized mice or primate models. Unfortunately, the cost of animal models has meant that studies of the HIV LTR in relevant animal models have been very limited (see Section 4.3). We also summarize the functions of the HIV auxiliary proteins Tat and Vpr in the control of HIV gene expression and latency. 

### 3.1. Pre-Initiation Complex (PIC) Formation and the General Transcription Factors (GTFs)

The formation of the pre-initiation complex (PIC) is an essential step leading to the recruitment of RNAPII at the core promoters of all class II genes. The PIC is formed by the stepwise assembly of a large complex composed of general factors of transcription (GTFs), including TFIIA, TFIIB, TFIID, TFIIE, TFIIF, TFIIH, the mediator complex and the RNAPII complex itself [179,180]. The first step in PIC formation is the recognition of the TATA box region by the TATA binding protein (TBP). TBP recognizes the TATA(A/T)(A/T)A(A/T)(A/G) specific sequence in the minor groove of the DNA located at approximately −30 of the transcription start site (TSS) (reviewed in [180,181]). Similar to canonical promoters, activation of the HIV requires the recruitment of TBP, although the recruitment of TBP is not sufficient for activation of the HIV LTR by the viral *trans*-activating protein Tat [182]. In the case of the HIV promoter, the TATA box has been shown to be recognized by TBP as an atypical “CATA” box sequence [183]. In typical promoters, TBP binds to the minor groove of the TATA box, inducing a DNA kink of 90°. The DNA–protein interaction is stabilized by the TFIIA protein following recruitment of TFIIB. These functions of TFIIA and TFIIB are thought to act on the HIV promoter, although unlike cellular promoters TFIIA and TFIIB function appears to be modulated by the viral Tat on the HIV promoter [184,185]. The HIV promoter can contact TFIIB, although it does not contain a consensus TFIIB recognition element (BRE) [186]. The TFIIF subunit RAP74 is required for Tat-activated HIV transcription and can compete with HIV Tat for binding to RNA polymerase II C-terminal domain (CTD) phosphatase FCP1 [187,188]. The general transcription factor TFIIH is required for HIV transcriptional activation [189], and the recruitment of TFIIH is important for the reactivation of latent HIV [190]. It is therefore clear that the formation of PIC upon the HIV LTR requires the majority of the canonical GTFs, and that the function of several PIC components can be modulated by HIV Tat. 

Although many canonical GTFs are required for PIC formation upon the HIV LTR, PIC formation is distinct from most cellular promoters, and these differences are important for the development of therapeutic molecules to selectively control HIV latency. For example, during canonical PIC formation, TBP is recruited to the promoter within a complex of 15 TBP-associated factors (TAFs) (reviewed in [191]). In contrast, a 2005 study suggested that PIC recruitment to the HIV promoter occurs with significantly less TAF occupancy than that observed at typical cellular promoters [192]. In fact, observations that the TATA box region of the HIV was specifically required for the *trans*-activation of the viral Tat protein (see Section 3.2.1) sparked the hypothesis that functionally specialized PIC, distinct from canonical PIC, might form upon the HIV LTR (reviewed in [173,193]). More recently, it was shown that an element within the core promoter region (−35 to −14) termed TASHET (TATA box and adjacent sequences of HIV essential for Tat *trans*-activation) is essential for the HIV LTR’s response to Tat (reviewed in [173]). Moreover, the specific capacity of TASHET to confer Tat-responsive transcription of the HIV viral promoter was shown to depend on CTGC DNA motifs flanking the HIV TATA box. Although it is now clear that the HIV core promoter is specifically recognized by host cell complex termed the pre-initiation complex of HIV (PICH), the identity of the proteins involved remains a mystery (Figure 1A). Since these complexes could represent new drug targets for HIV cure strategies, their identification is of high strategic value, as discussed in detail elsewhere (reviewed in [173]). 

#### 3.1.1. 7SK Complex (P-TEFb)

**Figure 2:**
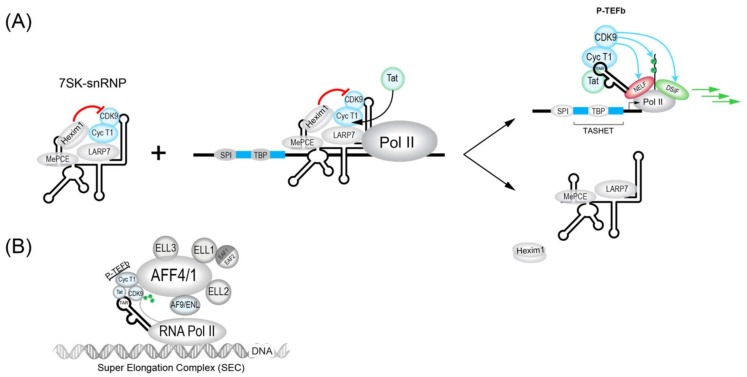
The role of the 7SK-snRNP/Tat/TAR in the activation of the HIV LTR is schematized. (**A**) The inactive HEXIM1-bound form of the 7SK-snRNP complex (left) forms upon the HIV LTR in its inactive state (middle). The HIV Tat *trans*-activator binds to Cyclin T1 to favor the recruitment of the active P-TEFb upon the HIV LTR (right top) and the release of an inactive form of 7SK-snRNP that lacks HEXIM1 (right bottom). Active P-TEFb phosphorylates the CTD of Pol II and other elongation factors to promote elongation of transcription. (**B**) A more detailed schematic view of Tat during activation of the HIV LTR in association with the super elongator complex (SEC). Tat plays a crucial role in the reactivation of HIV from latency and its action can be mimicked by several latency reversing agents (LRAs), such as JQ1.

During the initiation of transcription by RNAPII, the enzyme will be bound by the transcription factors NELF (Negative elongation factor) and DISF (DRB sensitivity-inducing factor), which will pause the transcription initiation [194,195] (Figure 2). To allow the paused polymerase to elongate, the 7SK complex, which is abundant in cells, plays a crucial role. The 7SK complex is a small nuclear ribonucleoprotein (snRNP) composed of a snRNA (small nuclear RNA) and the P-TEFb transcription factor itself is composed of a cyclin dependent kinase (CDK9) and cyclin T1. In addition, the proteins HEXIM1 and HEXIM2 will sequester a large part of CDK9 and inhibit its catalytic activity. The complex is stabilized by MePCE and LARP7 proteins (Figure 2) [196]. The 7SK complex also contains members of the so-called super elongator complex (SEC) including AFF4/MCEF, ENL/AF9 and ELL2 that also impact HIV transcription [197].

The dynamic association between snRNA 7SK and P-TEFb is also influenced by proteins such as bromodomain containing protein 4 (BRD4). BRD4 is key interaction partner for P-TEFb and can act to inhibit Tat *trans*-activation at the HIV LTR. In fact, BRD4 has been targeted pharmacologically by inhibitory molecules such as JQ1 [198,199,200] and MMQO [201] to antagonize BRD4 repression for the activation of HIV transcription (see Table 1). The chromatin regulatory protein HMGA1 also negatively modulates HIV transcription and can bind to both 7SK RNA [202] and TAR RNA [203]. Interestingly, the expression of TAR RNA alone can influence the expression of host cell 7SK-dependent mRNAs, implying potential reciprocal interactions between HIV 7SK complexes and HIV [194] that could influence viral latency. Ultimately, modulators of P-TEFb activity will impact its major roles the activation of processive HIV transcriptional elongation. These functions include the CDK9-dependent phosphorylation of NELF (negative elongation factor) and DSIF (DRB sensitivity-inducing factor), as well as in the phosphorylation of the carboxy terminal domain (CTD) of RNAPII (Figure 2) (reviewed in [197]).

HIV Tat recruits P-TEFb in association with the super elongator complex (SEC) to enhance RNAPII elongation. SEC complexes were found to contain either AFF4 (formerly Mcef1) or AFF1, in addition to AF9, ENL and ELL proteins (Figure 1 and Figure 2B) [204,205,206]. SEC is built upon a flexible scaffold of AFF4 or AFF1, with P-TEFb (Cycline T1 and CDK9), ELL family proteins (ELL1, ELL2, and ELL3), AF9, ENL, and EAF family proteins (EAF1 and EAF2) that can interact with HIV Tat (Figure 2B) [205,206,207,208]. The recruitment of SEC to chromatin-bound RNAPII can be facilitated via interactions with the PAF1 protein within the human Polymerase-Associated Factor complex (PAFc) [209]. As more detailed structural information on the SEC complex becomes available [207,210,211], the targeting of SEC components to therapeutically modulate HIV transcription should become more feasible. CRISPR-based functional studies suggest that ELL2 and AFF1 play key roles in HIV latency [212]. The targeting of SEC was shown by recent studies that have identified a chalcone derivative termed Amt-87 as a small molecule activator of latent HIV that acts by favoring the release of active P-TEFb from the 7SK snRNP [213]. Moreover, AFF4 fragments have been shown to inhibit HIV transcription in cultured cells [214], suggesting the possibility that targets within SEC could be used to shut down latent HIV for a functional cure (see Section 4.5).

In addition to their essential role in HIV transcriptional control, SEC complexes play a broad role in cellular gene expression. For example, transcriptomic studies in murine embryonic stem cells showed a role for SEC components at promoters with paused RNAPII, but also at certain unpaused promoters [215]. Interestingly, the Hox family of developmental transcription factors were found to be cellular targets for regulation by SEC, and their deregulation may underpin mixed lineage leukemia (MLL) driven by fusion proteins resulting from translocation of MLL to SEC genes such as AFF1, AFF4, ENL, and AF9 [215]. Mechanistically, SEC can be recruited to promoters via interactions with the mediator subunit MED26 [216] and components of the general transcription factor TFIID (notably TAF6) [217]. SEC complexes are intensively studied because of their role in HIV transcription and latency, but also due to their roles in other disease states including leukemia [218] and adenoviral replication [219]. There are currently three known SEC complexes that include SEC, the most well-studied complex, that acts upon the HIV promoter, as well as SEC-L2 and SEC-L3 [220]. These distinct SEC complexes contain different AFF family member scaffold proteins: AFF4/AFF1 for SEC, AFF2 for SEC-L2 and AFF3 for SEC-L3 [220]. Distinct SEC can differentially control elongation of genes, for example at the HSP70 promoter SEC, but not SEC-L2 or SEC-L3, is required for RNAPII release [220]. The cellular roles for SEC represent challenges for targeting this complex for an HIV cure, since it will be important to develop strategies specific to the HIV promoter avoiding off-target effects on of cellular SEC target genes.

#### 3.1.2. NF-κB

The NF-κB motifs within the HIV promoter are among the most studied elements of the HIV LTR and are thought to play a major role in inducible HIV gene expression. The NF-κB family of transcription factors form part of a major cell signaling pathway that controls cell survival and influences many cellular genes (for example, TRAF6, Inhibitors of Apoptosis (IAPS), and Bcl-2 family) (reviewed in [221]). The NF-κB family consists of five members: P50, P65, P52, c-Rel and ReIB (reviewed in [221]). The HIV-1 5′ LTR of all the major subtypes possess at least two cognate NF-κB binding sites around −104 to −80 called Core II upstream and Core I downstream separated by four nucleotides (reviewed in [222]). With the subtype C, the promoter contains 3–4 NF-κB sites.

The NF-κB pathway can be triggered by intracellular or extracellular stimuli that act by modulating the activation of protein kinase C (PKC). PKC is a potential drug target for the shock and kill strategy to cure HIV (see Section 4.4). NF-κB-activating stimuli can include interleukin 1 (IL-1), T-cell mitogens or pro-inflammatory cytokines such as TNFα. PKC protein will phosphorylate IκB kinase (IKK) to activate it. Upon activation, IKK phosphorylates the IκB protein in P65-P50-IκB complex. After phosphorylation, IκB will be ubiquitinylated and degraded by the proteasome, releasing P50-P65 to enter the nucleus and bind DNA. If P50-P50 dimers bind the NF-κB region upstream the TATA box, it can activate the HIV-1 transcription in cooperation with SP1 and other transcription factors (reviewed in [222]). In contrast, if P50-P65 is not present, the P50-P50 homodimer will bind the NF-κB sites and recruit CBF-1 that then recruits HDAC1 complexes to repress the promoter (reviewed in [222,223]). The HIV-1 promoter contains two copies of the same 10 pb repeat consensus NF-κB binding sites (reviewed in [222]) that are generally, although not universally [224], conserved in HIV isolates, underlining their importance in HIV transcriptional regulation in vivo. Several small molecules that activate the NF-κB pathway via PKC have been used to activate HIV transcription in the shock and kill HIV cure strategy, underscoring the potential clinical importance of the pathway (see Table 2). 

#### 3.1.3. NFAT1

The nuclear factor of activated T cells (NFAT1) is an important transcription factor of lymphocytes in regulating the homeostasis of the immune system [225,226]. One of the roles of NFAT1 is to be a key to the regulation of T cell differentiation in Th1, Th2, Th17, Tfh and Treg (T regulatory cells) [225]. The structure of NFAT1 has been determined by crystallography (residues 396–678) of the Rel homology region (RHR) of the protein bound to the κB site [226]. The protein is a dimer when bound to the κB motifs in the 5′ LTR of HIV (GGGACTTTCC), but remains a monomer in solution, suggesting that NFAT1 must bind DNA to form its dimeric interaction [226]. NFAT1 can also bind to distinct DNA elements, for example the TGGAATTTCCA motif from the IL-8 promoter [226]. Other NFAT family members (NFAT2, NFAT3 and NFAT4) have the same dimeric structure and are predicted to be also able to interact with the HIV promoter and therefore could also play similar roles in activating HIV transcription [226]. 

#### 3.1.4. SP1

SP1 (specificity protein 1) is important for basal activity of the HIV-1 LTR [227]. SP1 is generally considered to be constitutive in most cell types (reviewed in [223]). In the HIV-1 LTR, SP1 is localized between TATA box and NF-κB sites (reviewed in [228]). (Figure 1) In the majority of HIV-1 subtype, at least three SP1 sites are bound by three SP1 proteins and recruit co-factor COUP-TF interacting protein 2 (CTIP2) (reviewed in [223]). In certain subtype, the promoter contain 4–5 SP1 sites (reviewed in [223]). SP1 engages in multiple interactions such as with NF-κB bound to the LTR, interaction with YY1, protein–protein interaction with the PIC components and the distal SP1 monomers (reviewed in [222]). Furthermore, SP1 proteins are known to interact with TAF110 and be strictly necessary for the activation of the HIV promoter (reviewed in [222]). In another role, SP1/CTIP2 will cooperatively interact with YY1, NF-κB (P50-P50), SP3 and TFII-1 to recruit HDAC1, HDAC2 and HDAC3 to promote deacetylation of nucleosome 0 and 1 (nuc-0 and nuc-1). SP1/3 can then recruit CTIP2 with Suv39H1 to promote tri-methylation of histone 3 (H3) to create a deeper latency (reviewed in [223]). 

#### 3.1.5. RBF-2 (USF1/USF2/TFII-I)

A cellular factor, originally termed Ras-responsive region binding factor 2 (RBF-2), binds upstream of the NF-κB sites and immediately downstream of the HIV TATA box to its binding sites RBEIII and RBEI, respectively (Figure 1B). RBF-2 and RBF-1 were shown to be required for the response of the HIV LTR to signals from the tyrosine kinase/Ras/Raf pathway [229]. The full identity of RBF-1 remains to be determined, but it has been shown to contain subunits GABP that bind to the RBEIV/ETS motif at positions −151 to −142 of the HIV LTR [230]. Importantly, RBEIII is a highly conserved motif [231], implying a significant role in HIV replication. Subsequent work has shown that RBF-2 is composed of at least three subunits, including USF1, USF2 and TFII-I [232,233]. Furthermore, an intact RBEIII RBF-2 binding site was shown to be critical for the activation of the HIV LTR by T cell activation [234]. Based on the above-described observations, RBF-2 (USF1/USF2/TFII-I) has emerged as a key factor in the regulation of HIV latency [228,235]. 

### 3.2. Viral Auxiliary Proteins

#### 3.2.1. Tat

In addition to host cell transcription factors, the virally encoded proteins play a major role in HIV transcription and therefore latency. As mentioned above, the viral Tat *trans*-activating protein plays a central role in forming a feed-forward regulatory loop that accelerates HIV transcription via stimulation of both the initiation and the elongation of transcription [192,236]. Tat interacts directly with the nascent stem-loop RNA structure at the 5′ end of all viral RNAs to extract the active form of p-TEFb for viral transcription (Figure 2). Although HIV transcription is regulated by T cell signals, the Tat circuitry also results in stochastic fluctuations in HIV expression, a phenomenon proposed to contribute to evasion of the host cell immune response [192,236]. In addition to its direct impact on HIV transcription, Tat can be secreted by infected cells without lysis [237]. Tat has also been implicated in activating the NF-κB pathway [238], inducing apoptosis and the inhibition of siRNA formation by Dicer (Review in [239]).

#### 3.2.2. Vpr

The Vpr (Viral protein R) protein also has multiple roles in the regulation of cellular processes to favor HIV replication. Among these roles, Vpr impacts the nuclear import of the pre-integration complex into the nucleus, blocks the cell cycle in the G2/M phase to increase viral transcription and participates in the induction of apoptosis and *trans*-activates the HIV-LTR and host cellular genes (Review in [239]). In the case of Vpr’s impact on HIV transcription, the effect is largely indirect, acting primarily through the HIV TATA box region [150] and possibly also through the upstream elements NF-κB and AP1 [240].

## 4. HIV Cure/Remission Strategies

Timothy Brown, formerly referred to as the Berlin Patient, underwent stem-cell transplantation in 2007 to treat his acute myeloid leukemia. The stem-cells were chosen from a donor that had a homozygous 32 base pair deletion in the HIV coreceptor CCR5 gene (delta32CCR5), and the outcome of the intervention is that Mr. Brown has been living without ART treatment with no viral rebound since [241]. Timothy Brown’s story is an inspiration to all people living with HIV. For scientists, it provides the proof of principle that HIV can be cured. Unfortunately, the stem-cell transplantation procedure is not broadly applicable clinically due to its high mortality rate. Although Timothy Brown is the only case of an HIV cure, some individuals that received early ART treatment and were later found to naturally control HIV viremia in the absence of ART, showing that HIV remission can occur [242]. The term cure generally refers to the eradication of HIV from the patient, while the term remission, or functional cure, is most often used to describe the clinical outcome wherein the patient can live with low or undetectable virus in the absence of ART for prolonged periods without adverse impacts on disease status. In both cases, the objective is to remove the need for ongoing ART, the risk of transmission, and the stigmatization associated with HIV status. The documentation of a cure and natural cases of remission, although coming from a very small number of patients, have spurred efforts to achieve a cure or remission of HIV.

### 4.1. CRISPR/Cas9

**Figure 3:**
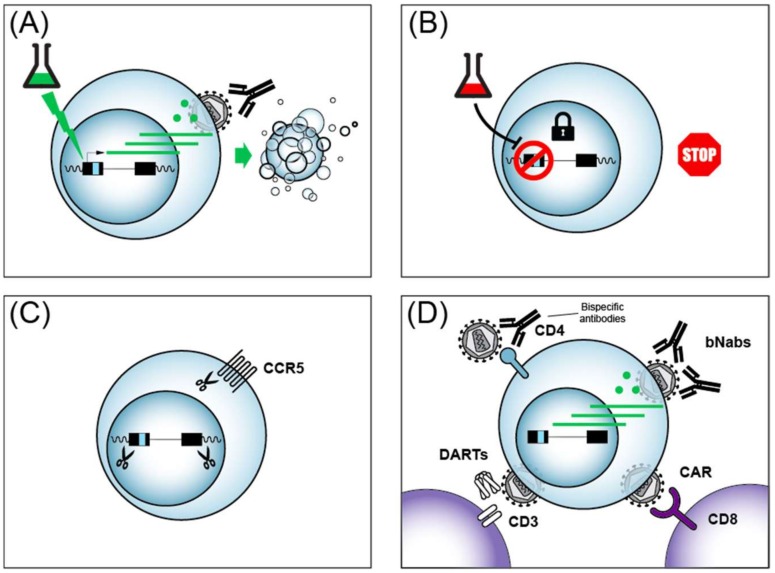
Four major strategies for an HIV cure or remission. (**A**) The shock and kill strategy to purge viral reservoirs is illustrated. See text for details. (**B**) The block and lock strategy for a functional cure with HIV in deep latency is illustrated. See text for details. (**C**) Genome editing by CRISPR/Cas9 to excise the HIV provirus or edit essential host dependency factors (e.g., CCR5). (**D**) Immunotherapy strategies to enhance the host cell immune response against HIV. CD4+ helper lymphocytes are in blue and CD8+ cytotoxic lymphocytes are in purple.

As detailed above, transcription of the HIV provirus is the first step and a rate-limiting step in the reactivation from latency. Therefore, most cure/remission strategies will require the therapeutic manipulation of HIV transcription to attack the HIV reservoir. One exception to this rule are gene editing approaches that use the CRISPR/Cas9 system. CRISPR (Clustered Regularly Interspaced Short Palindromic Repeats) were discovered in 1987 [243], triggering the rapid development of genome editing tools now based on CRISPR Cas9. The editing exploits the *Streptococcus pyogenes* Cas9 protein to introduce clustered regularly interspaced short palindromic repeats with a guide RNA target. CRISPR/Cas9 can, in principle, be used to excise the HIV provirus from the genome or to host cell genes essential for HIV propagation, such as the CCR5 co-receptor (Figure 3C). Early studies raised concerns that HIV can rapidly become resistant to CRISPR/Cas9 [244], however it appears that using multiple guide RNAs to target HIV may overcome resistance [245]. Several obstacles currently lie in the way of the clinical use of CRISPR/Cas9 including the long-term safety, off-target effects, and ethical issues surrounding genome editing. Importantly, it is unclear how the CRISPR guide RNA, along with the Cas9 protein might be efficiently delivered to all reservoirs including difficult to reach tissues such as the brain or testicles. Nonetheless, CRISPR/Cas9 remains an active area of pursuit for potential future strategies to achieve an HIV cure or remission (reviewed in [246]).

### 4.2. Immunotherapies

HIV infection is initially followed by a strong reduction in viremia due to the host immune response [247]. CD8+ lymphocytes have been shown to play an important role in the suppression of HIV using Simian Immunodeficiency Virus (SIV) infection in the rhesus monkey primate model [248]. The mechanisms of CD8+ cell control of HIV viremia involves direct cytotoxicity via perforin and granzyme secretion, as well as less well characterized non-cytotoxic activities (reviewed in [249]). Importantly, CD8+ cells are also required for suppression of SIV viremia and HIV [250,251], supporting the use of immunotherapy as a clinical strategy to aid in the elimination of HIV reservoirs for a cure or remission.

One gene therapy approach targeting CD8+ cells being explored towards an HIV cure includes chimeric antigen receptor (CAR)-modified immune cells that have been engineered to target HIV (reviewed in [252,253]). CAR T cells can be engineered to express T cell receptors (TCRs) that interfere with HIV replication after genetic engineering of autologous CD8+ CTL (Figure 3D). Strategies that have been investigated in HIV cure research include CARs that target HIV because the TCR contains a segment of the HIV receptor CD4, or engineering TCR that contact HIV via single-chain fragment variable (scFv) from antibody segments from broadly neutralizing antibodies (bNAbs). Early clinical trials with CAR T cells failed to reduce HIV levels because the CARs were eliminated by cytotoxic T-lymphocyte responses of patients [254], underscoring the complexity of such approaches. More recently, Hematopoietic Stem/Progenitor Cell (HSPC) gene therapy showed a more sustained CAR delivery in non-human primates [255]. Strategies to enhance anti-HIV CAR activity include the addition of signaling moieties to the engineered TCRs, such as the intracellular domains of the lymphocyte signaling molecules CD28 or CD3, and have shown encouraging anti-HIV activity with patient cells ex vivo (reviewed in [252]). The success of CAR T cell treatment of other diseases such as acute lymphoblastic leukemia (ALL) [256], provides indirect hope that such approaches might one day be applicable to people living with HIV.

Much progress has been made in recent years identifying broadly neutralizing antibodies (bNAbs) against HIV from the B-cells of people living with HIV, and these antibodies could add to the arsenal of molecules for cure strategies (Figure 3D) (reviewed in [257]). bNAbs can be delivered to patients via infusion safely [258]. In addition to their ability to neutralize multiple strains of HIV, bNAbs have been shown to impart an unanticipated enhancement of CD8+ T cell-mediated immunity in macaque monkeys [259]. A caveat of bNAbs is that the use of a single bNAb can lead to the selection of viral escape mutations. Recently, the combined use of two bNAbs was shown to prevent viral rebound in people infected with antibody-sensitive HIV in the absence of ART [260]. bNAbs thus represent another class of cure/remission tools, likely to be used in conjunction with additional cure strategies, in the quest to eradicate HIV.

Another cure strategy that builds on the high specificity of bNAbs are bispecific antibodies that recognize HIV epitopes, but are also designed with scFvs that recognize functional proteins of the immune system with the hope of enhancing the immune response against HIV (Figure 3D) (reviewed in [261,262]). Such bispecific reagents can be used to neutralize to HIV, as shown by bispecific antibodies against HIV envelope protein (Env) and CD4 [263]. Bispecific antibodies are of particular interest in cure research, since they could potentially impact latent HIV reactivation and be immune effectors at the same time. In this vein, bispecific antibodies directed against HIV Env and the CD3 T cell co-receptor were shown to activate both latent HIV and target cell lysis in vitro [264]. Careful design optimization of bispecific antibodies continues to improve their potency and breadth [265,266]. More recently, trispecific antibodies that target against HIV were designed, underscoring the flexibility of such molecules [267]. Reagents termed Dual-Affinity Re-Targeting Molecules (DARTs) are antibody derivatives analogous to bispecific antibodies (Figure 3B), and also show promise in the recruitment of cytotoxic lymphocytes via the CD3 receptor for the killing of HIV infected CD4 cells [268,269]. Multi-specific antibodies and their derivatives have thus emerged as promising agents in the quest for an HIV cure/remission. 

### 4.3. HIV Transcription and Immune Evasion: A Game of Cat and Mouse?

HIV evolved in the face of immune responses, and the hardwired latency program it encodes has presumably been selected for since it provides the advantage of evasion of the host immune system and therefore viral persistence. It is only an unfortunate consequence of HIV latency that the latent virus also evades current antiretroviral drugs, all of which only act on their targets during active viral replication. The mechanisms that permit HIV to escape host immune surveillance are limited access of antibodies to Env, antigenic variation due to high mutation rates, downregulation of the immune recognition proteins MHC I and CD4 at the cell surface by HIV Nef, the crippling of CD4 help due to the elimination of CD4+ cells, and the capacity of the virus to undergo latency and reactivation (reviewed in [270,271]). Of these mechanisms, perhaps the contribution of latency remains the most enigmatic. It is a game of “cat and mouse” between the immune system and HIV in which the immune system appears to have the upper hand, since viremia is initially suppressed. Virtually nothing is known about the details of this game of cat and mouse, but eventually HIV (mouse) succeeds in winning in 99% of cases, with only approximately 0.5–1% of infected individuals that control viremia in the absence of ART, termed elite controllers [272].

**Figure 4:**
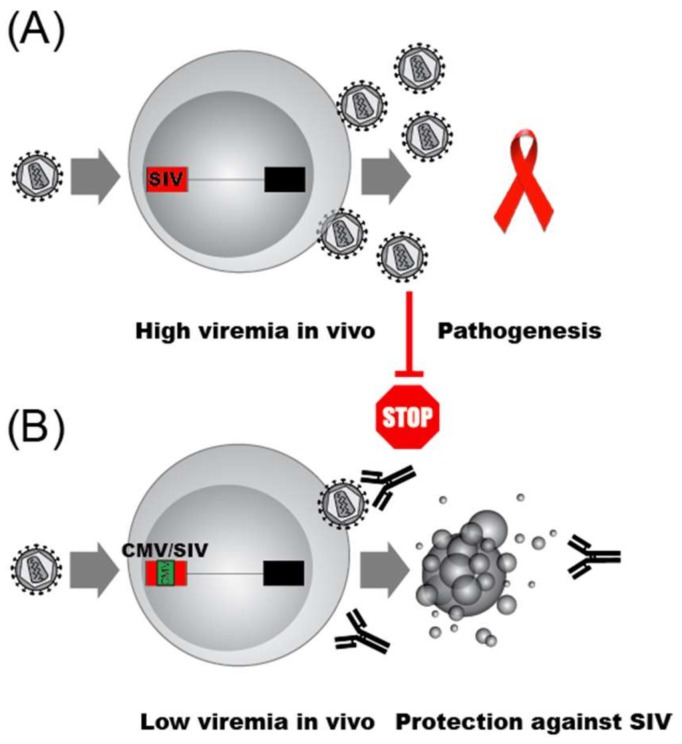
The essential role of the SIV LTR in immune evasion is depicted. (**A**) A virus containing the wild type promoter infects to produce high viremia levels and AIDS-like disease in macaque monkeys. (**B**) A virus with a chimeric promoter, in which a portion of the SIV promoter has been replaced by a fragment of the CMV promoter, is replication competent but produces the same peak viral output as the parental SIV in cultured cells, but low level viremia in macaque monkeys. Animals infected by a virus whose expression is driven by the SIV/CMV swapped promoter achieve immune clearance of the SIV/CMV chimeric virus and are then protected against infection by the wild type SIV virus.

As the HIV promoter and the transcription factors that bind it control HIV latency, and latency itself is thought to have evolved as a means of immune evasion, the impact of promoter function on viral clearance is an important yet underexplored question. An elegant set of experiments using SIV infection of macaque monkeys and promoter exchange experiments have provided the first glimpses of the impact of promoter structure on immune clearance [273]. Infection of macaques with wild type SIV resulted in high viremia AIDS-like pathogenesis (Figure 4A). In stark contrast, the replacement of a segment of the SIV promoter that included the NF-κB and SP1 sites, as well as the SIV TATA box, with a roughly analogous portion of the CMV promoter resulted in infectious virus that produced low viremia and no disease in macaques (Figure 4B). Importantly, both the chimeric SIV/CMV promoter chimeric virus and the parental SIV promoter virus, produced equivalent peak levels of virus after infection of human T lymphocyte cell lines or primary PBMCs, although the SIV/CMV virus displayed delayed replication kinetics. The immunological parameters that explain the impact of promoter architecture on clearance of replicating virus remain obscure. It is noteworthy that the parental SIV virus generated stronger activation of CD4+ and CD8+ lymphocytes than the chimeric promoter containing virus as judged by increases in the number of CD69+ positive T cells. Nevertheless, the observed serum antibody responses and cellular immunity against HIV epitopes (e.g., Gag, Nef) from both the CD4+ and CD8+ compartments generated in animals infected with the parental SIV versus the SIV/CMV viruses appeared comparable. Most strikingly, not only were the animals that received the SIV strain with an exchanged promoter healthy, but they were protected against challenge with the parental SIV virus. In contrast to the promoter swapping studies of Blancou et al., one of the only other studies of the SIV promoter in rhesus monkeys showed that deletion or substitution of the NF-κB and SP1 motifs resulted in a virus that retained the ability to cause AIDS-like disease [274]. The results provide compelling evidence implicating the architecture of the 5′ LTR in determining whether SIV infection results in disease or clearance by the host immune system, and drive home the urgency of more intense investigation into the role of promoter architecture in the search for both a cure and a vaccine for HIV.

Although it is not known how the promoter exchange uncoupled HIV from its immune evasion capabilities, it is likely that not only steady state transcription rates were impacted. The chimeric SIV/CMV promoter responds weakly to the viral *trans*-activator Tat, perhaps since the TATA box region is essential for LTRs activation by Tat (reviewed in [173]). Similarly, HIV Vpr acts through the elements of the SIV promoter that were exchanged in the SIV/CMV [150]. How the viral promoter allows HIV immune escape remains completely unknown. One purely speculative conceptual framework we evoke is that the very rapid response of the virus to extracellular cues from host immune cells permits at least some virus-infected cells to avoid killing, analogous to the way a boxer avoids being punched by ”slipping” the opponents oncoming blow, before counterpunching. Evidence that the speed of viral replication versus the speed of the T cell infiltration can impact viral escape has been reported for SIV [275]. We note as a possible player in immune escape that CD8+ cells are thought to inhibit HIV transcription (reviewed in [249]), although the possible mechanisms responsible remain to be fully defined [276,277]. One thing is clear: Further investigation into how the specific architecture of the HIV promoter allows the virus to evade immune clearance is important to achieve not only an HIV cure, but also an effective vaccine. 

### 4.4. Shock and Kill

One strategy to eliminate latent reservoirs of HIV from infected people that has to date garnered the lion’s share of effort and resources has been called the shock and kill (or kick and kill) approach that has been extensively reviewed [278,279,280,281]. The approach is conceptually straight forward, and involves the activation of latent HIV so that the virus can then be eliminated by ART treatment, almost certainly in conjunction with immunotherapy (Figure 3A). Much progress has been made in the identification of latency reversing agents (LRAs) that act on host cell transcription factors to induce HIV gene expression. The most common LRAs act through three main mechanisms: (i) NF-κB activation; (ii) HDAC inhibition; or (iii) mimic HIV Tat by driving the formation of the active form of P-TEFb from its inactive 7SK snRNP-bound form. For brevity, we present a summary of several common LRAs and their targets in Table 1. Although LRAs, or combinations thereof, indeed activate HIV expression in vitro and in vivo, clinical trials to date have failed to show any decrease in the viral reservoirs (reviewed in [280]) by shock and kill, even when used in combination with HIV vaccines [282]. There are many avenues that remain to be explored in the optimization of the shock and kill approach, yet there are daunting obstacles to overcome as well. In particular, recent work shows that LRAs activate HIV expression from not more than 5% of latently infected primary CD4+ cells, even when used in combination [73]. Another hurdle to overcome to achieve efficient shock and kill, is the recently reported observation that the treatment of HIV infected people with LRAs such as the HDAC inhibitor romidepsin only increased early HIV transcription initiation and elongation, without significant impacts on later events such as transcriptional completion and multiple splicing [283]. These findings imply that later steps in HIV gene expression in addition to transcription will have to be targeted for effectively purging HIV reservoirs. Although the shock and kill cure approach remains a highly active area of research, the preponderance of evidence provided to date underscores the strategic importance of also pursuing alternative ways to achieve an HIV cure/remission. 

### 4.5. Block and Lock

A strategy distinct from the shock and kill HIV cure approach that is drawing increasing attention is a remission strategy termed block and lock (Figure 3B). The term block and lock has been coined relatively recently to describe a strategy to induce a deep and permanent state of HIV latency to achieve a functional cure (remission) for HIV [289]. The idea of inhibiting HIV transcription to develop anti-HIV drugs predates the ART era (reviewed in [290]), but it is only recently that long sought inhibitors of HIV Tat have been discovered. The small molecule didehydro-Cortistatin A (dCA) binds to HIV Tat to inhibit Tat-mediated *trans*-activation [291]. Recently, it has been shown that dCA binds selectively to the unstructured basic region of Tat to prevent the Tat–TAR RNA interaction [292]. dCA prevents viral reactivation from patient CD4+ cells ex vivo [293]. Even more encouragingly, dCA treatment slows viral rebound after ART withdrawal in a humanized mouse model [289]. dCA has therefore emerged as an appealing latency promoting agent (LPA) for block and lock remission. The dCA target Tat has been shown to counteract the cleavage of TAR into small RNAs that are proposed to feed back to negatively regulate the HIV LTR via epigenetic changes [294]. Therefore, recent advances in the understanding of the role of cellular proteins involved in TAR processing may provide a rich source of potential drug targets for novel block and lock treatments that impact the epigenetic state of the LTR [295]. In addition to dCA, another LPA showing promise is ABX464, a small molecule that interacts with Cap Binding Complex (CBC) to prevent HIV Rev-mediated nuclear export of unspliced HIV transcripts [296]. ABX464 was shown to block viral rebound after ART removal in humanized mice, and more recently was also reported to be well tolerated in humans [297]. ABX464 is currently in clinical trials for the treatment of ulcerative colitis, and a recent next generation transcriptome sequencing suggests it may act by enhancing the splicing of HIV [298]. Building on these findings, we anticipate an acceleration of efforts to improve existing LPAs, identify novel LPAs from natural sources, chemical screening and rational design (reviewed in [299]), as well as the testing of LPA in animal models and clinical trials. Recently, curaxin 100 [300] and levosimendan [301] were reported as new potential LPAs that inhibit HIV transcription. Table 2 summarizes potential small molecule LPAs and their impacts on HIV.

The block and lock strategy has several potential advantages over the shock and kill strategy. Firstly, while clinical trials to date suggest that ART interruption does not represent a major health risk, the possibility of adverse effects with viral reactivation are difficult to rule out. In contrast to shock and kill, the block and lock strategy inhibits HIV reactivation, therefore avoiding safety concerns related to increased HIV replication. Secondly, the development of block and lock reagents, should they fail to achieve a functional cure, would still be of clinical value since these drugs would represent new antiretrovirals that could be used in the case of drug resistance. A third advantage, which remains hypothetical, is that, unlike LRAs, LPAs may uncouple HIV gene expression from immune system cues (e.g., CD8+ CTLs), enhancing immune clearance. The prediction of this hypothesis can be tested in primate models, in which the efficiency of immune clearance of SIV is monitored in response to LPA treatments.

In addition to small molecule approaches to block and lock, gene therapy approaches are being explored. For example, shRNAs that target the HIV LTR were recently shown to induce epigenetic silencing and block reactivation of HIV in cultured cell models of HIV latency [302]. In another approach, HEXIM1-Tat chimeric peptides were reported to block HIV reactivation in cell lines [303]. While there is currently no efficient way to deliver shRNAs or peptides to HIV reservoirs in patients, these findings could pave the way to improved LPAs in the future. In summary, recent progress in development of block and lock strategies have generated a growing optimism that such a strategy could be used in the future to bring about a functional cure for HIV.

### 4.6. Other Cure/Remission Strategies

In addition to the strategies described above, fresh approaches are continually being conceived and tested in the quest for a cure/remission. One of these strategies includes the use of HIV-dependent suicide gene therapy vectors to kill cells of the latent reservoir [305]. A second alternative strategy is the selective killing of latently infected cells with oncolytic viruses, a method that has shown encouraging results in cultured cells [306]. A third alternative approach is the use of Smac mimetics to reverse HIV latency [307] and selectively induce death of latently infected cells [280]. A fourth notable approach to deplete the latent reservoirs entails the use of small molecule inhibitors of HIV Nef function to enhance the CD8 T cell-mediated elimination of latently infected cells [308]. Finally, we note more than one of the cure/remission strategies could be combined to synergistically achieve a functional cure in the clinic.

## 5. Conclusions and Perspectives

It is not yet clear which cure/remission strategies will be the most effective in achieving an HIV/AIDS free world. Along the inexorable but difficult pathway to a cure/remission, it is worth being reminded of the lessons learned from earlier hard-fought victories against HIV/AIDS. Firstly, members of the HIV infected population must be included at all stages of the fight, from advocacy to the conception and analysis of biomedical studies and clinical trials [309]. Secondly, despite our zeal to rapidly move cure/remission strategies into the clinic, we must not lose sight of the critical gaps in our biological knowledge of HIV. Lifesaving ART was only made possible once basic science gave us an intimate knowledge of the drug targets and their role in the HIV life cycle. Thirdly, and most importantly, HIV/AIDS is a global problem and any cure/strategy that will succeed must be able to be implemented in resource-limited areas of the world.

Here, we have highlighted the growing interest in the block and lock strategy to achieve an HIV remission. We suggest that small molecules would be an affordable and scalable way to achieve block and lock, and that such drugs may also potentially cripple the virus’ ability to escape host immune surveillance. Finally, we point to the gap in our knowledge of the cat and mouse-like interaction between the immune system and HIV that underpins HIV persistence as a crucial area for investigation in the coming years.

The goal of achieving an HIV cure or remission is important for the 36.9 million people currently living with HIV, but also as a means to definitively halt the HIV pandemic for future generations. Furthermore, should a cure and/or remission for HIV be discovered, it would at last open the way to curing other pathogenic infections that depend on latency for their persistence, such as herpes viruses (e.g., HSV1, HSV2, and EBV) (reviewed in [310]), chronic viral pathogens that have yet to emerge, and perhaps even latent bacteria, such a *Mycobacterium tuberculosis* (reviewed in [311]). 

## Figures and Tables

**Table 1:** A list of characterized HIV latency reversal agents (LRAs).

Latency Reversing Agent (LRA) *	LRA Class (Target/Pathway)	Reference (s)
Valproic acid	HDAC inhibitor	[284]
SAHA	HDAC inhibitor	[285]
Romidepsin	HDAC inhibitor	[286]
JQ1	BET domain inhibitor/BRD4	[199]
MMQO	BET domain inhibitor/BRD4	[201]
Prostratin	Stimulates PKC/NF-κB	[287]
BIX01294	Methyltransferase inhibitor	[288]

* Note that to date clinical trials with LRAs have not shown a significant reduction in viral reservoirs (see text).

**Table 2:** A list of potential HIV latency promoting agents (LPAs).

Latency Promoting Agent (LPA)	LPA Mode of Action	Animal Model Data	Clinical Trial Data
dAC	Binds HIV tat to inhibit transcription [292]	Represses viral rebound in BLT humanized mice [289]	N.A.
ABX464	HIV Rev inhibitor [296]; HIV splicing enhancer [298]	Delayed viral rebound in NOG humanized mice [297]	Good safety and tolerability; measurable reduction in HIV DNA [304]
Curaxin (CBL0100)	FACT inhibitor [300]	N.A.	N.A.
levosimendan	Inhibits HIV transcription [301]	N.A.	N.A.

N.A., not available.

## Supplementary Materials

The following are available online at https://www.mdpi.com/1999-4915/11/3/269/s1, Table S1: A list of miRNAs putatively involved in the regulation of HIV-1 latency.
