# Supplementary material for: Cat and Mouse: HIV Transcription in Latency, Immune Evasion and Cure/Remission Strategies
Source: Viruses. 2019 Mar 18;11(3):269. doi: 10.3390/v11030269 (PMC6466452; doi:10.3390/v11030269)
Supplement: Supplementary file 1 [file viruses-11-00269-s001.pdf]

Table S1. A list of miRNAs putatively involved in the regulation of HIV-1 latency

	Target(s)	miR	Effect(s) on HIV	Model(s)	Reference(s)
HIV	3′LTR	miR-133b, miR-138, miR-149, miR-326	Impair HIV replication	CD4+ cell lines, primary CD4+ T cells	[1]
	U3/3′UTR	miR-382-5p, miR-28-5p	Impair HIV replication	CD4+ cell lines, primary T cells, monocytes/macrophages	[2,3]
	U3/5′LTR/ negative responsive element	miR-N367 *	Impairs HIV promoter activity	CD4+ T cell lines	[4]
	3′ UTR/ Nef	miR-29a, miR 29b, miR-1290, miR-196b, miR-223-3p, miR-326	Impair HIV replication	Non lymphocytic cell lines and PBMC	[1–3,5–8]
	Env	miR-125b-5p, miR-150-5p, miR-133b, miR-138-5p, miR-28 and miR-382,	Promote latency	CD4+ cell lines, primary $CD4^+$ T cells	[1,2]
	Vif	miR-155-5p	Impairs HIV replication	CD4+ cell lines, primary $CD4^+$ T cell	[9]
	Pol	miR-92a-3p	Impairs HIV replication	CD4+ cell lines,	[1]
	Gag	miR-149-5p, miR-423-3p	Impair HIV replication	CD4+ cell lines, primaryCD4+ T cell	[1,9]
	5′ LTR (TATA box)	miR-H3 *	Promotes viral replication	Activated primary CD4+ T cells	[10]
	HDAC-1 mediated chromatin remodeling of 5′LTR	TAR-derived miR *	Impair transcription	CD4+ cell lines and PBMC	[11]

	Target(s)	miR	Effect(s) on HIV	Model(s)	Reference(s)
Host cell factors	Cyclin T1	miR-198	Impairs HIV replication	Cell lines, primary monocytes/macrophages	[12]
	Cyclin T1	miR-29b, miR-150, miR-223	Impair HIV replication	Primary resting CD4+ T cells	[13]
	Cyclin T1	miR27b	Impairs HIV replication	Primary resting CD4+ T cells	[13]
	PCAF	miR-17/92 ; miR17-5p and miR20a	Impair HIV infection	CD4+ cell lines, primary CD4+ T cell	[14]
	p21	Let7c, miR-124a	Promote replication	CD4+ cell lines	[15]
	TASK1	miR-34a-5p	Promote replication	CD4+ cell lines	[15]
	SIRT-1	miR-217, miR-34a	Promote HIV-1 expression	Epithelial cell lines, CD4+ T cell lines	[16,17]
	SAMHD1	miR-155	Promotes transcription	Astrocytes	[18]
	SAMHD1	miR-181a	Promotes replication	Astrocytes	[18]
	Pur-α	miR-15a/b, miR-16, miR-20a, miR-93, miR-106b	Impair HIV-1 replication	Primary monocytes	[19]
	MeCP2	miR-132	Promotes replication	CD4+ cell lines, primary CD4+ T cell	[20]

* Indicates that the miRNA is encoded by the HIV-1 virus

1. Houzet, L.; Klase, Z.; Yeung, M.L.; Wu, A.; Le, S.-Y.; Quiñones, M.; Jeang, K.-T. The extent of sequence complementarity correlates with the potency of cellular miRNA-mediated restriction of HIV-1. *Nucleic Acids Res.* **2012**, *40*, 11684–96.

2. Huang, J.; Wang, F.; Argyris, E.; Chen, K.; Liang, Z.; Tian, H.; Huang, W.; Squires, K.; Verlinghieri, G.; Zhang, H. Cellular microRNAs contribute to HIV-1 latency in resting primary CD4+ T

lymphocytes. *Nat. Med.* **2007**, *13*, 1241–7.

3. Wang, X.; Ye, L.; Hou, W.; Zhou, Y.; Wang, Y.-J.; Metzger, D.S.; Ho, W.-Z. Cellular microRNA expression correlates with susceptibility of monocytes/macrophages to HIV-1 infection. *Blood* **2009**, *113*, 671–4.

4. Omoto, S.; Fujii, Y.R. Regulation of human immunodeficiency virus 1 transcription by nef microRNA. *J. Gen. Virol.* **2005**, *86*, 751–5.

5. Ahluwalia, J.K.; Khan, S.Z.; Soni, K.; Rawat, P.; Gupta, A.; Hariharan, M.; Scaria, V.; Lalwani, M.; Pillai, B.; Mitra, D.; et al. Human cellular microRNA hsa-miR-29a interferes with viral nef protein expression and HIV-1 replication. *Retrovirology* **2008**, *5*, 117.

6. Nathans, R.; Chu, C.-Y.; Serquina, A.K.; Lu, C.-C.; Cao, H.; Rana, T.M. Cellular microRNA and P bodies modulate host-HIV-1 interactions. *Mol. Cell* **2009**, *34*, 696–709.

7. Sun, G.; Li, H.; Wu, X.; Covarrubias, M.; Scherer, L.; Meinking, K.; Luk, B.; Chomchan, P.; Alluin, J.; Gombart, A.F.; et al. Interplay between HIV-1 infection and host microRNAs. *Nucleic Acids Res.* **2012**, *40*, 2181–96.

8. Wang, P.; Qu, X.; Zhou, X.; Shen, Y.; Ji, H.; Fu, Z.; Deng, J.; Lu, P.; Yu, W.; Lu, H.; et al. Two cellular microRNAs, miR-196b and miR-1290, contribute to HIV-1 latency. *Virology* **2015**, *486*, 228–38.

9. Whisnant, A.W.; Bogerd, H.P.; Flores, O.; Ho, P.; Powers, J.G.; Sharova, N.; Stevenson, M.; Chen, C.-H.; Cullen, B.R. In-depth analysis of the interaction of HIV-1 with cellular microRNA biogenesis and effector mechanisms. *MBio* **2013**, *4*, e000193.

10. Zhang, Y.; Fan, M.; Geng, G.; Liu, B.; Huang, Z.; Luo, H.; Zhou, J.; Guo, X.; Cai, W.; Zhang, H. A novel HIV-1-encoded microRNA enhances its viral replication by targeting the TATA box region. *Retrovirology* **2014**, *11*, 23.

11. Klase, Z.; Kale, P.; Winograd, R.; Gupta, M. V; Heydarian, M.; Berro, R.; McCaffrey, T.; Kashanchi, F. HIV-1 TAR element is processed by Dicer to yield a viral micro-RNA involved in chromatin remodeling of the viral LTR. *BMC Mol. Biol.* **2007**, *8*, 63.

12. Sung, T.-L.; Rice, A.P. miR-198 inhibits HIV-1 gene expression and replication in monocytes and its mechanism of action appears to involve repression of cyclin T1. *PLoS Pathog.* **2009**, *5*, e1000263.

13. Chiang, K.; Sung, T.-L.; Rice, A.P. Regulation of cyclin T1 and HIV-1 Replication by microRNAs in resting CD4+ T lymphocytes. *J. Virol.* **2012**, *86*, 3244–52.

14. Triboulet, R.; Mari, B.; Lin, Y.-L.; Chable-Bessia, C.; Bennasser, Y.; Lebrigand, K.; Cardinaud, B.; Maurin, T.; Barbry, P.; Baillat, V.; et al. Suppression of microRNA-silencing pathway by HIV-1 during virus replication. *Science* **2007**, *315*, 1579–82.

15. Farberov, L.; Herzig, E.; Modai, S.; Isakov, O.; Hizi, A.; Shomron, N. MicroRNA-mediated regulation of p21 and TASK1 cellular restriction factors enhances HIV-1 infection. *J. Cell Sci.* **2015**, *128*, 1607–16.

16. Zhang, H.-S.; Wu, T.-C.; Sang, W.-W.; Ruan, Z. MiR-217 is involved in Tat-induced HIV-1 long terminal repeat (LTR) transactivation by down-regulation of SIRT1. *Biochim. Biophys. Acta* **2012**, *1823*, 1017–23.

17. Zhang, H.-S.; Chen, X.-Y.; Wu, T.-C.; Sang, W.-W.; Ruan, Z. MiR-34a is involved in Tat-induced

HIV-1 long terminal repeat (LTR) transactivation through the SIRT1/NFκB pathway. *FEBS Lett.* **2012**, *586*, 4203–7.

18. Pilakka-Kanthikeel, S.; Raymond, A.; Atluri, V.S.R.; Sagar, V.; Saxena, S.K.; Diaz, P.; Chevelon, S.; Concepcion, M.; Nair, M. Sterile alpha motif and histidine/aspartic acid domain-containing protein 1 (SAMHD1)-facilitated HIV restriction in astrocytes is regulated by miRNA-181a. *J. Neuroinflammation* **2015**, *12*, 66.

19. Shen, C.-J.; Jia, Y.-H.; Tian, R.-R.; Ding, M.; Zhang, C.; Wang, J.-H. Translation of Pur-α is targeted by cellular miRNAs to modulate the differentiation-dependent susceptibility of monocytes to HIV-1 infection. *FASEB J.* **2012**, *26*, 4755–64.

20. Chiang, K.; Liu, H.; Rice, A.P. miR-132 enhances HIV-1 replication. *Virology* **2013**, *438*, 1–4.
